# A Haptic Feedback Scheme to Accurately Position a Virtual Wrist Prosthesis Using a Three-Node Tactor Array

**DOI:** 10.1371/journal.pone.0134095

**Published:** 2015-08-11

**Authors:** Andrew Erwin, Frank C. Sup

**Affiliations:** 1 Mechanical Engineering Department, Rice University, Houston, TX, United States of America; 2 Mechanical and Industrial Engineering Department, University of Massachusetts Amherst, Amherst, MA, United States of America; University of Chicago, UNITED STATES

## Abstract

In this paper, a novel haptic feedback scheme, used for accurately positioning a 1DOF virtual wrist prosthesis through sensory substitution, is presented. The scheme employs a three-node tactor array and discretely and selectively modulates the stimulation frequency of each tactor to relay 11 discrete haptic stimuli to the user. Able-bodied participants were able to move the virtual wrist prosthesis via a surface electromyography based controller. The participants evaluated the feedback scheme without visual or audio feedback and relied solely on the haptic feedback alone to correctly position the hand. The scheme was evaluated through both normal (perpendicular) and shear (lateral) stimulations applied on the forearm. Normal stimulations were applied through a prototype device previously developed by the authors while shear stimulations were generated using an ubiquitous coin motor vibrotactor. Trials with no feedback served as a baseline to compare results within the study and to the literature. The results indicated that using normal and shear stimulations resulted in accurately positioning the virtual wrist, but were not significantly different. Using haptic feedback was substantially better than no feedback. The results found in this study are significant since the feedback scheme allows for using relatively few tactors to relay rich haptic information to the user and can be learned easily despite a relatively short amount of training. Additionally, the results are important for the haptic community since they contradict the common conception in the literature that normal stimulation is inferior to shear. From an ergonomic perspective normal stimulation has the potential to benefit upper limb amputees since it can operate at lower frequencies than shear-based vibrotactors while also generating less noise. Through further tuning of the novel haptic feedback scheme and normal stimulation device, a compact and comfortable sensory substitution device for upper limb amputees might be created.

## Introduction

In the United States, one out of every 520 people have an upper limb amputation [1]. While prosthetics have been in existence since ancient times, the human-prosthetic interface has remained largely unchanged until the advent of myoelectric prosthetics in the 1960s [2]. For a myoelectric prosthetic user, efferent signals on the residual limb are used to control the prosthesis; however, the prosthetic device does not compensate for the loss of afferent signals [3, 4]. This requires the amputee to rely on vision alone for precise control of the prosthesis reducing the effectiveness and speed at which it can be operated [5, 6]. As a result, haptic feedback devices have been explored as a means to restore sensory feedback for amputees.

The need for sensory feedback in prosthetic devices was discussed by Shannon in the 1960s when he identified the main sources for creating stimulation [7–9]. While the means to create stimulation have remained relatively unchanged, advances in technology have allowed for more efficient and compact devices, such as piezoelectric actuators, DC eccentric rotating mass motors, and linear resonant actuators, to be used in wearable haptic applications. Additionally, advancements in control electronics and surface electromyography (sEMG) electrodes have increased the potential for haptic devices to be incorporated into a prosthesis, although such a device is yet to be commercialized [10]. Several approaches to providing haptic feedback for prosthetic users have been proposed including continuous grasp force feedback through varying a single vibrotactor’s frequency [11], continuous elbow joint rotation through skin stretch [6], and eight hand positions through an eight node array of vibrotactors using on/off feedback [12].

The importance of incorporating a sensory substitution device in a prosthesis is becoming increasingly more important as more functional hand prostheses are being developed with multiple degrees of freedom (DOF) [4, 10] as compared to 1DOF basic gripper prostheses [2, 9]. Currently, multi-DOF prosthetics are controlled 1DOF at a time through sEMG control on the residual limb. Many DOFs can be controlled at the same time through targeted reinnervation surgery by rerouting neurons from the residual limb to other areas of the body [13, 14] and it has been suggested that the rerouting of the afferent neurons could also be used to provide more realistic afferent feedback. However, a study using haptic feedback with an individual who had undergone targeted reinnervation surgery showed that the feedback was difficult to apply since the feedback was applied at the same location where efferent signals were measured. It has been shown that haptic feedback has a reduced or obviated benefit when applied to moving muscles [15]. Thus, most studies have focused on providing haptic feedback through stimulating the forearm or upper arm of the residual limb [6, 16–18].

This paper presents the implementation of a novel haptic feedback scheme on a haptic feedback prototype device which generates stimulations normal (perpendicular) to the skin at low-frequencies. Compared to previous approaches, this approach offers an intuitive and compact way of providing rich haptic information to the user through only a few tactors. The objective of the haptic feedback scheme is to relay angular position information of a virtual wrist prosthesis so that the visual demand required to operate the prosthesis is reduced. The haptic feedback scheme is novel since it uses normal stimulation, relays angular positions through discrete modulation of tactor frequency, and uses three tactors to intelligently and intuitively encode the virtual wrist positions. This paper also compares the use of the normal stimulation to vibratory shear stimulation. The comparison is made since normal stimulation is often cited as being ineffective compared to shear, but normal stimulation could have important ergonomic advantages over shear since it can operate at lower frequencies and generates less noise. Also note that the scope of the scheme and device is not limited to relaying wrist positional information, but could easily be used to encode other information. For example, it could encode pressure information such as touch, grip force [11], orientation information to a pilot [19], or end-effector position to someone teleoperating a robot [20].

In this paper, the background section covers the typical means of generating shear and normal stimulation as well as describing the studies which claim normal stimulation to be ineffective. The next section details the design and specifications of the novel normal stimulation haptic prototype device while the experimental design section describes the experimental setup for a study where participants use haptic feedback to control a 1DOF virtual wrist prosthesis without visual feedback. The haptic feedback mapping section explains the novel haptic feedback scheme and is followed by the results section which presents statistical analyses comparing the angle targeting errors obtained from the normal stimulation device, shear-type vibrotactors, and no feedback cases. The paper concludes with the presentation and discussion of the results.

## Background

### Mechanical Stimulation—Shear

There are two types of mechanical stimulation: shear and normal. Shear has been widely used in the literature and most notably through vibrotactors which create shear vibrotactile stimulation. Vibrotactile stimulation is the use of a vibratory stimulator that stretches the skin, causing vibration on the surface of the skin. Vibrotactile stimulation is most often created through the use of vibrotactors (e.g. coin motors), but can also be created through piezoelectric or linear resonant actuators. Although these small coin motors are ubiquitous in the literature, they do have limitations. Controlling the frequency of the tactors is difficult since it is easily affected by pressure [21] and the surface waves that travel from them can still have 10% of the original amplitude after traveling 6 cm across the surface of the skin [22]. As a result of this, the C2 tactor was designed to reduce these surface waves [16], but it comes at an increased cost, size, and weight compared to coin motors.

Skin stretch is another form of shear stimulation which provides stimulation by stretching the skin translationally or rotationally [6, 17]. In a targeted motion study of a virtual prosthetic arm by Bark et al., the effectiveness of skin stretch vs. vibrotactile stimulation on the forearm was compared. Vibrotactile feedback was applied using a C2 tactor and skin stretch through a benchmark device. Feedback for the tactor was a continuous mapping of amplitude to virtual position, whereas the skin stretch device modulated twist of the end effector to virtual position. Since the skin stretch device was bidirectional, it was able to split the virtual workspace in half for each direction of arm rotation. Results from the experiment indicated that skin stretch was the superior feedback device and that participants learned open loop strategies throughout the experiment, due to improvement from no feedback trials at the beginning to the end of the study [16]. Using skin stretch is limited by sufficient contact area of the end effector to avoid causing pain to the user, and the increased size and weight of the devices compared to vibrotactors.

### Mechanical Stimulation—Normal

Normal stimulation, which has been created in the literature through motors [17, 18] and voice coils [23], was declared by Shannon in 1974 to be ineffective compared with vibrotactile stimulation, although his reasoning was purely qualitative [7]. A quantitative study of shear vs. normal stimulation was performed by Biggs. His study indicated that normal stimulations require more force and displacement than tangential stimulations at the forearm to match the intensity of a 1.5 mm normal displacement on the forearm [24]. However, the reference stimulation in the study was kept as a normal stimulation reference with a constant displacement. A tangential reference along with more displacements should have been used. Another concern is what the results mean for vibratory stimulations since the study was only performed with slow moving reference stimulations. One notable configuration using normal stimulation was created by Antfolk et al.; the device consists of five actuators placed in the orientation of an open palm on the forearm driven by servo motors which could depress a plastic button into the skin for stimulation. Antfolk et al. show the potential of using a device that creates stimulation at more than one sight so that each stimulator could map to a finger. Disadvantages of the device were its large power consumption and noise from the motors [18].

## Normal Stimulation Voice Coil Prototype Design

Devices using normal stimulation are relatively unexplored in the literature. This may be due to Shannon and Biggs who declared shear stimulation to be more effective than normal stimulation [7, 24]. While their claims are sound, they have limitations and do not necessarily mean that normal stimulation is less effective than shear stimulation for feedback schemes that were not addressed in their work. This paper looks to restore tactile and proprioceptive sensations using normal stimulation. The objective of the study is to implement a feedback scheme on a prototype device created for laboratory use to relay position feedback of a wrist prosthesis. The haptic feedback device has not been optimized for prosthetic use, but was rather created as a concept for relaying normal stimulation to the skin. As such, accuracy in interpreting stimulations from the device might be improved with optimization of the prototype.

**Fig 1:**
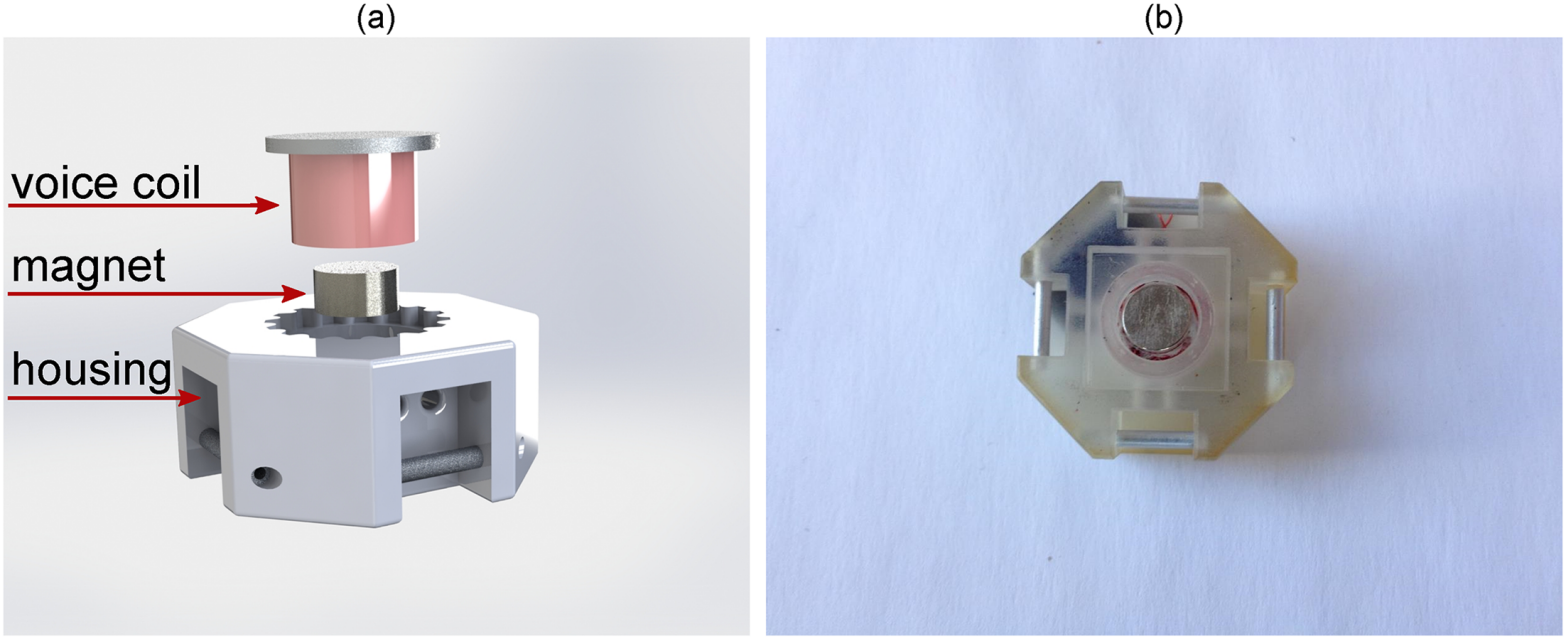
Wearable voice coil assembly. (a) Rendered exploded view and (b) bottom view.

The normal stimulation tactor shown in Fig 1 is modular, easily reconfigurable, and expandable. Details of the design are presented in [25]. The voice coil based design uses a magnet to repulse against the skin and was used for its compactness and light weight as opposed to using motors. A neodymium magnet (5233 Gauss Surface Strength, 6.35 mm × 3.18 mm) is used in the voice coil to maximize force, while minimizing size and electrical resistance. A custom 3D printed ABS plastic shell houses the voice coils and modules are connected to each other with Velcro. The final specifications for the modules are 25 × 25 × 11.5 mm, 7.6 g, 4.3 Ω, and can produce 0.29 N at 1 A.

Prior to the study detailed here, the effectiveness of this device was evaluated where participants were asked to differentiate between the five stimulation sites [25]. Participants wore the haptic feedback device in a cross pattern with 5 mm center-to-center spacing on the ventral side of the forearm and lay their arm on a table with palm facing up. Participants reported 86% correct responses in differentiating between the 5 individual stimulation sites, which was greater than the recognition between stimulation from five servomotors on the forearm by Antfolk et al. [18].

## Experimental Design

An experiment was designed to test the efficacy of using normal stimulation haptic feedback to control a 1DOF virtual wrist prosthesis. The virtual wrist was controlled using surface electromyography (sEMG) electrodes as used in myoelectric prostheses. The objective of the study was to examine participants’ errors in angle targeting tasks without visual feedback using the voice coil device and compare it to cases of no feedback and vibrotactile feedback.

### Experimental Setup

#### Participants

Eight able-bodied participants with no sensory impairments, 4 male and 4 female with a mean age of 23 (range 20–31, *σ* = 3.5), volunteered to participate in the study. The duration of the experiment varied between 60–90 minutes for each participant. Approval for the experiment was obtained through the University of Massachusetts Amherst Institutional Review Board.

#### Virtual Wrist Workspace and Equipment

**Fig 2:**
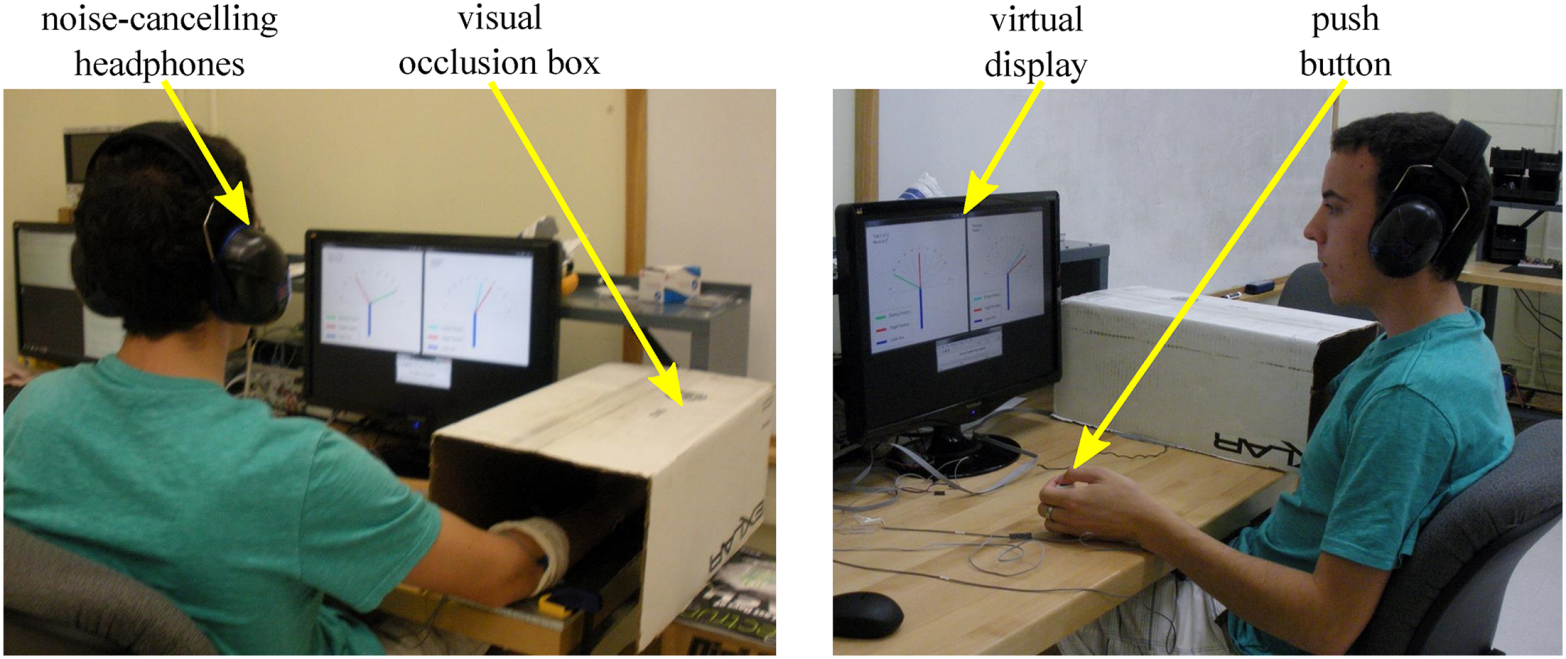
Illustration of the experimental setup used in the study. The individual in this manuscript (as outlined in the PLOS consent form) has given written informed consent to publish these case details.

The virtual wrist was given 1DOF to model wrist flexion and extension of an able-bodied person with a range of motion of ±90° serving as the workspace for this study. All targets in the study were between ±60° so the full range of motion allowed for overshoot of the extreme targets. Although flexion and extension in able-bodied persons is typically less than ±90°, a wrist prosthesis could be made to have this range of motion. Participants were equipped with two sEMG electrodes (Motion Lab Systems MA411 Surface EMG preamplifier), one placed on the ventral side of the forearm and one on the dorsal side to control the flexion and extension movements respectively. Both were placed proximal to the elbow and a ground electrode was placed at the head of the ulna on the wrist. Participants controlled the virtual wrist prosthesis through flexing and extending their own wrist. During trials, participants loosely gripped a handle fixed to the desktop to reduce proprioception during the experiment. By gripping the handle participants could produce an sEMG signal without the need to significantly rotate their wrist. During feedback trials, participants wore noise canceling headphones (Tasco’s Nextera Over-the-Head Earmuff) and additionally an air conditioning unit provided background noise to block audio cues from the tactors. During trials with haptic feedback, visual cues from the tactors were eliminated by having the participant’s arm placed under a box. To signal the end of a trial participants were given a button to press. The experimental setup is shown in Fig 2.

#### Calibration

At the start of each session, the participants’ offset and maximum comfortable flexion and extension sEMG signals were calibrated. First, each participants’ resting sEMG data was recorded for 3 seconds while resting their hand on the motion limiting handle. The offsets were subtracted from their respective signals. The second calibration was to record maximum flexion and extension signals from the participants by instructing participants to exert 80–90% of their maximum force for 3 seconds, a force they would be comfortable applying during the experiment. The mean of each signal was recorded and used to determine the digital gains of each signal. Gains were found by dividing 5.5 by the maximum signals so that the maximum input to the virtual wrist was 5 units since a 0.5 unit deadband was used as part of the virtual wrist’s dynamics.

### Virtual Wrist Dynamics and sEMG Filtering

MATLAB Simulink Real-Time and a National Instruments PCI-6229 DAQ were used to simulate the virtual wrist dynamics and for recording experimental data. A digital sampling frequency of 1.0 kHz was used. An analog filtering circuit recommended by Motion Lab Systems was used for the pre-amplified electrodes. In addition the sEMG signals were subtracted from a calibrated offset, filtered digitally with a 20 Hz first order high pass filter; rectifier; and 2 Hz first order low pass filter [26], and digitally amplified. The flexion and extension signals were then compared, and the greater signal was sent into the virtual wrist dynamics. The virtual wrist dynamics consisted of a deadband of 0.5 units to simulate static friction, a 5 unit saturation, and 2nd order transfer function

$$\frac{\Theta (s)}{V(s)}=\frac{1}{Js^{2}+bs}$$ (1)

where *J* = 0.5 is the virtual wrist inertia, and *b* = 2.5 is the damping [6].

## Haptic Feedback Mapping of Virtual Wrist Position

**Fig 3:**
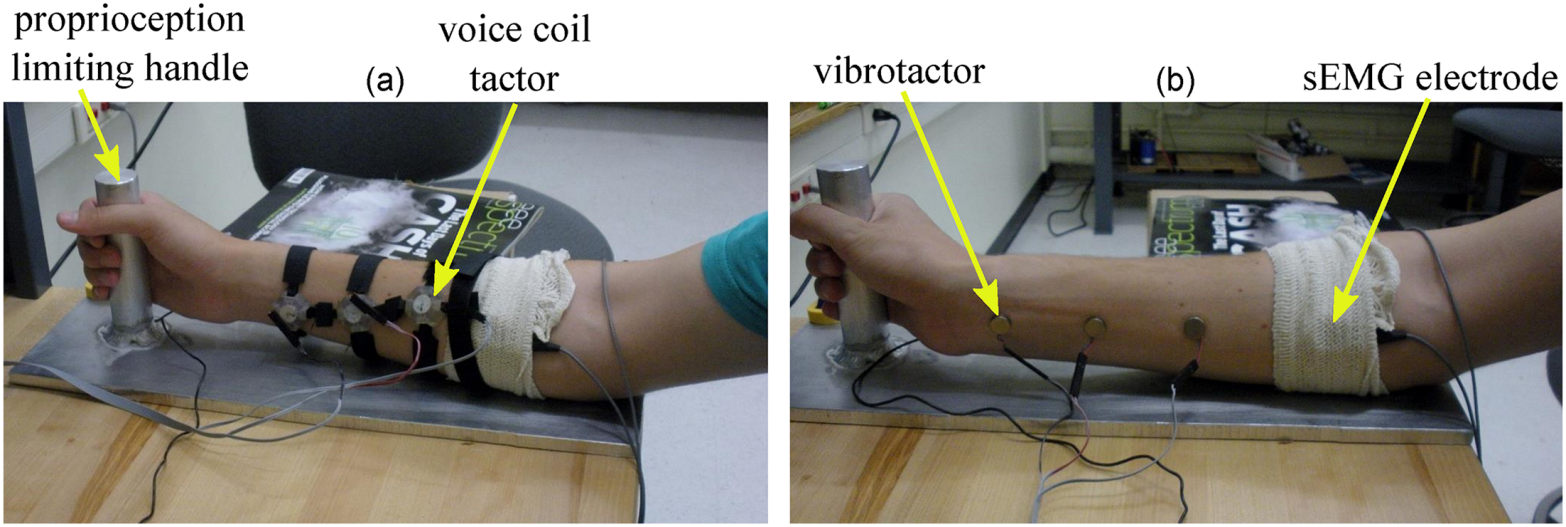
(a) Position of voice coil tactors, and (b) vibrotactors on the forearm during experimentation. The figure shows the motion limiting handle and placement of one of the sEMG electrodes.

During trials with haptic feedback, participants used a three-node array of either voice coil actuators or vibrotactors (Fig 3). The vibrotactors used in the study were Solarbotics VPM2 which vibrates at 200 Hz (±50 Hz) at 3 V and 80 mA. They were placed on the ventral side of the forearm, with the top actuator being placed just a few centimeters from the hand and then the other two were subsequently placed with 5 cm center-to-center spacing towards the elbow. The spacing was chosen to minimize total length of the array while remaining above the approximately 4 cm two-point threshold on the forearm [27].

Both the voice coils and vibrotactors used the same novel feedback mapping from virtual wrist position to stimulation, but with different operating frequencies. Each device had four discrete frequencies at which it could operate and were found by fitting a log curve from the minimum frequency of stimulation to the maximum since humans can more easily recognize log changes than linear ones [16]. For the voice coils, the minimum frequency was determined to be 5 Hz and 100 Hz was chosen as the maximum and the four levels are represented by the equation

$$f\approx 5*10^{0.433x}$$ (2)

where *f* is the frequency of stimulation and *x* is an integer between 0–3. In addition the voice coils were operated at 0.9 A with duty cycles of 10, 20, 30, and 50 percent in order of increasing frequency level. For the vibrotactors, the voltage through the tactor was controlled to modulate frequency. The minimum applied voltage was such that the tactors vibrated just enough for detectable sensation and the maximum frequency was based on a voltage that the tactors could operate continuously and the voltages are given by

$$f\approx 1.5*10^{0.159x}.$$ (3)

**Fig 4:**
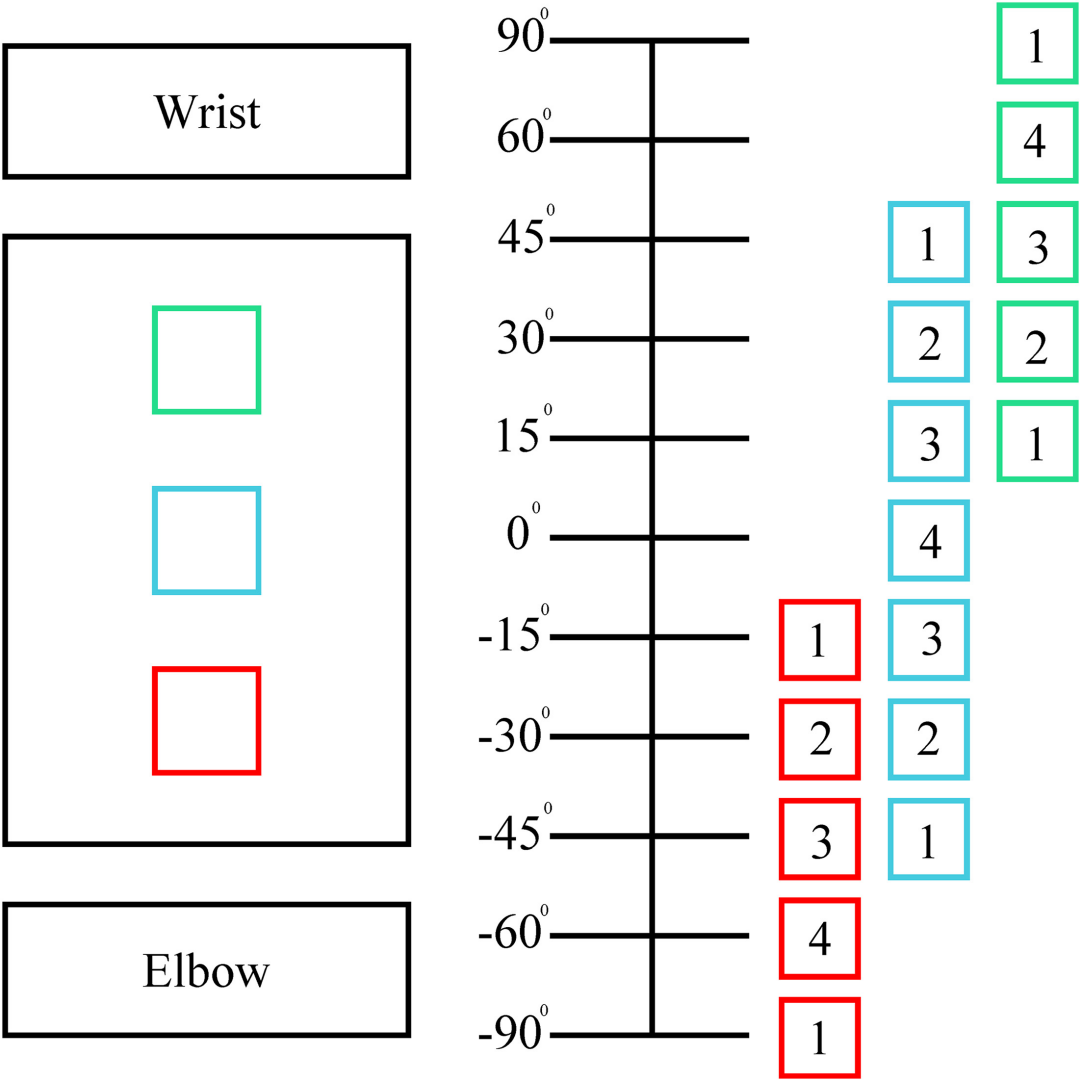
Feedback schematic presented to participants. The green, blue, and red boxes to the left of the angle marking line show the positions of the tactors on the forearm. The boxes to the right of the line show the frequency amplitude (values in Table 1) of the tactors at a given virtual wrist position.

**Fig 5:**
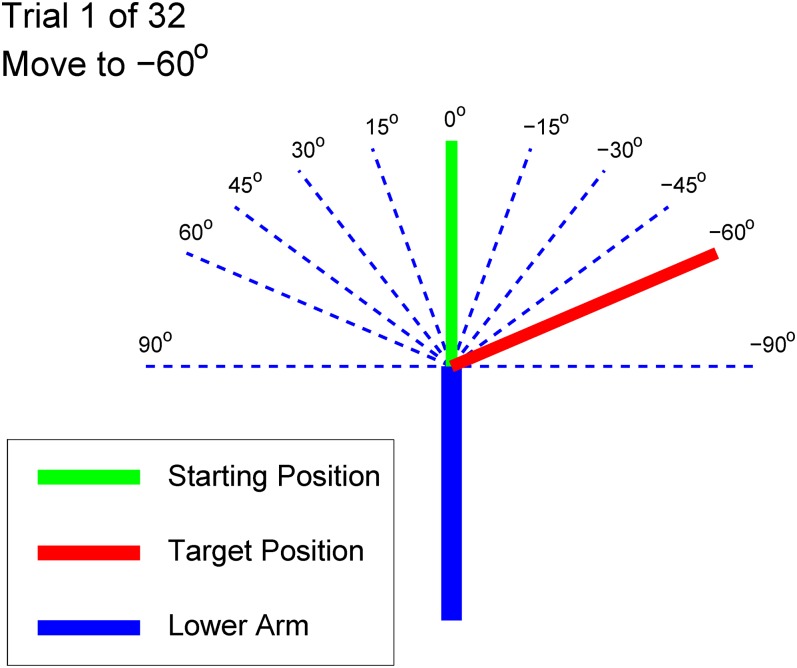
Visual display shown to participants at the beginning of an experiment. A similar display was shown at the end of the trial except that the Starting Position annotation and line was replaced with a Result annotation and line.

The feedback schematic and operating characteristics for the tactors can be seen in Fig 4 and Table 1, respectively. Each tactor was activated independently or in combination to indicate 11 discrete wrist positions, covering the ±90° workspace (Fig 5). Since the feedback was discretized, feedback was on for a given unit of ±7.5° in the ±60° operating range. For example, if the virtual wrist was between 52.5° to 67.5°, the top tactor would be operating at maximum frequency.

**Table 1:** Feedback control characteristics.

	Frequency Level	1	2	3	4
Voice Coils	Frequency (Hz)	5	10	50	100
	Duty Cycle (%)	10	20	30	50
Vibrotactors	Voltage (V)	1.5	2.2	3.1	4.5

### Angle Targeting Trials

#### Practice Sessions

Prior to recorded trials, participants had 8 practice trials for no feedback and the two types of mechanical feedback. For the no feedback practice session, participants could control and watch the virtual wrist prosthesis move in real-time. This allowed the participants to make a visual connection of their input sEMG signals to movement of the virtual wrist. They were allowed to practice this for three 30 s periods and then they performed the 8 practice trials. For the feedback cases, they were shown the same screen up to three times, but instead of them moving the arm, the wrist display moved on its own from -90° to 90° so the participants could link the feedback sensations to wrist position. They were also shown a diagram of the mapping of feedback to virtual wrist position to aid in their understanding the feedback scheme (Fig 4). The objective of the practice sessions was to familiarize participants with controlling the wrist and the feedback patterns, but not to become experts in either.

#### Known Initial Wrist Angle

For known initial wrist angle trials, participants were given an initial wrist position and a target position to move the wrist to. Initial positions started at either -60°, 0° or 60°. Participants were instructed to move to targets anywhere from -60° to 60° separated by 15° increments (9 total targets). Movements were constrained such that the target was at least 45° from the starting position making a total of 16 combinations for the trials. Participants were instructed to complete the trials as accurately as possible, but were not given a time limit. Participants underwent four sets of trials: No Feedback 1 (NF1), Voice Coil and Vibrotactile Feedback, and then No Feedback 2 (NF2). Each participant first performed NF1, and then half the participants were randomly assigned to having Voice Coil Feedback presented next while the other half were assigned Vibrotactile Feedback. All participants ended with NF2. By randomly assigning half the participants to having Vibrotactile Feedback second and half to having Voice Coil Feedback, learning effects for participants could be equalized. Each participant underwent 16 trials in each no feedback case and 32 trials for the feedback cases. This experimental design was chosen to be similar to that used in [6] to enable comparing results with their study as well as other similar ones controlling a virtual limb with the use of haptic feedback. However, in the study of Wheeler et al. only three targets were used and we elected to increase the amount to nine to make a more realistic study as well as examine how having more targets affected the results.

#### Unknown Initial Angle

In addition to the 32 trials of known initial angles with feedback, participants performed 10 more trials in the feedback cases where they did not know the initial wrist angle. These trials were performed directly after the known initial angle trials were completed for the given feedback type. The objective of these trials was to determine if participants could determine their initial position from the feedback and then move to the target accurately. Since without feedback this would not be possible, it was not included in these trials. Trials were such that starting positions were constrained to -60°, 0° or 60° and ending positions were either -60°, -30°, 0°, 30° or 60°. Both Vibrotactile Feedback and Voice Coil Feedback had two trials where the target position was the same as the starting position to test the participant’s ability to recognize that the wrist was in the correct position without needing to move it.

## Results

### Known Initial Angle Trials

#### Angle Targeting Accuracy

**Fig 6:**
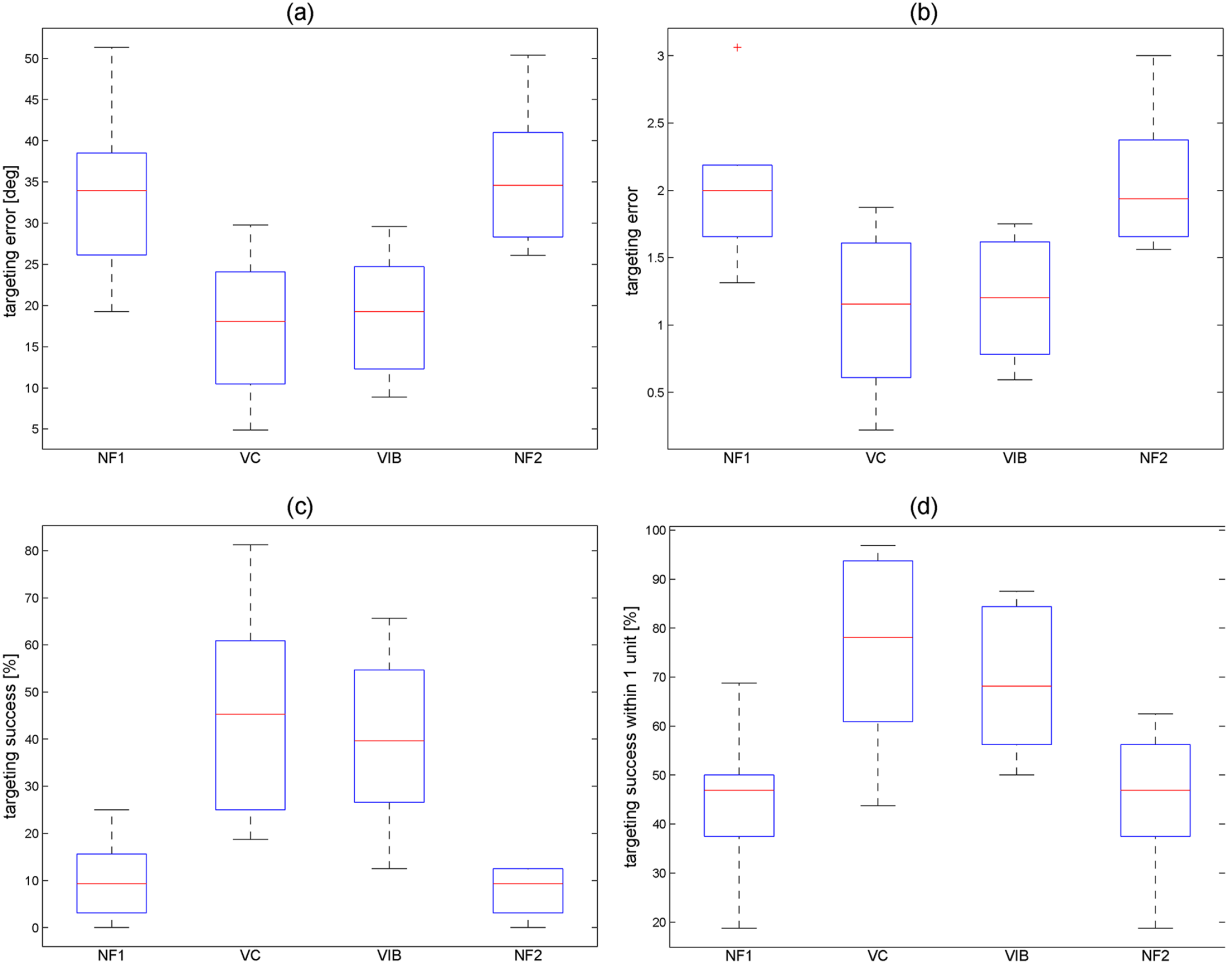
(a) Box plots of participants’ mean continuous targeting errors, (b) mean discretized targeting errors, (c) success in reaching the target and (d) near success in reaching the target for the No Feedback #1 (NF1), Voice Coil (VC) Feedback, Vibrotactile (VIB) Feedback, and No Feedback #2 (NF2) cases. The solid line in the boxes shows the median and the edges of the boxes are the 25th and 75th percentiles. The whiskers extending from the boxes show the most extreme data not considered to be outliers (within ±2.7*σ*) and “+” markers denote outliers.

The result of most importance to the study was the comparison of average absolute targeting error (absolute value of the final position subtracted by the target position) for each participant with the four types of feedback. In the remainder of the paper targeting errors will refer to absolute targeting errors. The range of mean angle targeting errors for participants were 19.3°-51.3°, 4.9°-29.8°, 8.9°-29.6°, and 26.1°-50.4° for NF1, Voice Coil Feedback, Vibrotactile Feedback, and NF2 respectively. In addition to examining targeting errors in degrees, they can also be expressed in discretized units for the 11 unit workspace where each angle marking on Fig 5 is a unit of workspace and corresponds to the 11 different feedback sensations. A box plot of average participant continuous and discretized targeting errors can be seen in Fig 6 and the range of mean discretized feedback errors for Voice Coil and Vibrotactile Feedback were 0.22–1.88 and 0.59–1.75 units respectively. A one-way repeated measure analysis of variance (ANOVA) was performed on participants’ mean targeting errors for all feedback cases which showed a significant difference in means, *F*(3,21) = 11.8, *p* = 9.5*10^−5^. Mean completion time of the trails can be seen in the following list: NF1 = 3.93 s (*σ* = 1.68), Voice Coil = 12.52 s (*σ* = 7.84), Vibrotactile = 10.28 s (*σ* = 5.98), and NF2 = 3.84 s (*σ* = 1.87). Additionally average success and near success rate of reaching the target were of interest. Reaching the target is defined as a trial where the wrist ended on the correct unit and near success is defined as the ending position being ±1 unit from the target. Box plots of participants’ average and near success are shown in Fig 6. A one-way repeated measure ANOVA of success showed a significant difference in means, *F*(3,21) = 21.9, *p* = 1.1*10^−6^, and similarly for near success *F*(3,21) = 13.6, *p* = 3.8*10^−5^.

For the three repeated measures performed, post-hoc pairwise comparisons were performed with Bonferroni corrections. With Bonferroni corrections the new significant alpha value for comparisons was 8*10^−3^. For all three cases (mean targeting errors, success, and near success), NF1 compared to NF2 (*p* > 0.47) and Voice Coils compared to Vibrotactile (*p* > 0.36) showed no significant differences. Also for all three cases Voice Coils were significantly different (*p* < 2.4*10^−3^) than NF1 and NF2, and Vibrotactile Feedback was significantly different (*p* < 2.6*10^−3^) than NF2. However, Vibrotactile compared to NF1 was only significant different (*p* = 2*10^−3^) in the success trials and not significantly different than NF1 in mean targeting errors (*p* = 9.6*10^−3^) and near success (*p* = 1.1*10^−2^). Note that Vibrotactile cases compared to NF1 that were not significant were a result of using the conservative Bonferroni correction. This correction was applied since it was used in other similar studies [6, 12] but the comparisons might be significant if a less conservative correction was applied.

#### Step Size and Ending Position Effects

**Fig 7:**
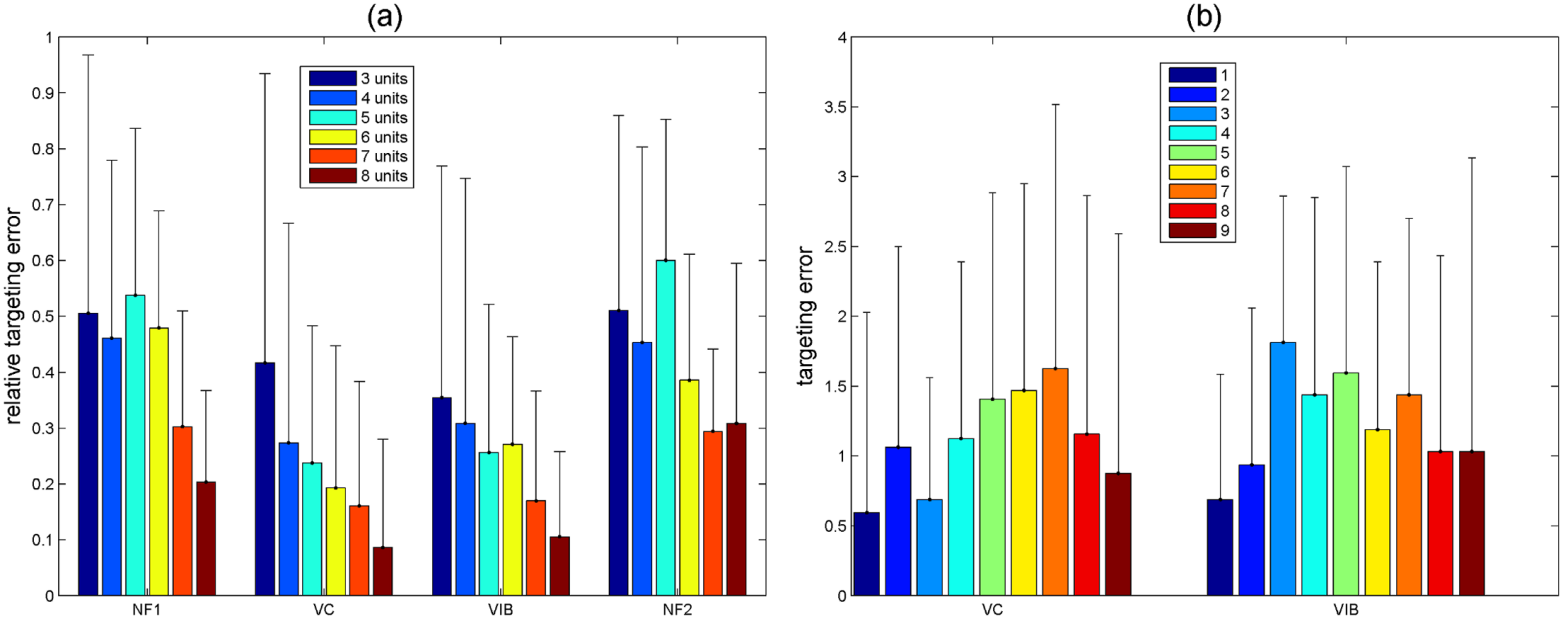
(a) Bar chart of relative targeting error for all possible step sizes (distance between initial and target positions) for the NF1, Voice Coil (VC) Feedback, Vibrotactile (VIB) Feedback, and NF2 cases. Legend shows the step sizes in discretized units (3–9 units = 45°–120°). (b) Bar chart of targeting error based on the ending (target) position for the Voice Coil (VC) and Vibrotactile (VIB) Feedback cases. Legend shows the ending position in terms of discretized units (1–9 units = 60° to -60° virtual wrist positions).

Also of interest to the study was if the results were dependent on step size and ending position. Step size is defined here as the distance between the initial wrist position and target position for that trial. Bar charts for relative targeting error (absolute targeting error/step size) based on step size and error depending on ending position can be seen in Fig 7. The No Feedback case is not included in this figure since there is no difference in its performance for varying ending positions.

#### Learning in the Study

The effect of order of feedback device presentation was examined by comparing mean errors of participants who had a given device first to those who had it second. This was done for both feedback modes using a one-way ANOVA which showed no statistical significance in either comparison. Learning within trials was examined by fitting a linear regression line for each participant across their trials. The regressions indicated that only one participant for NF1 and Voice Coil feedback had a statistically significant negative slope and one participant from Voice Coil and one from Vibrotactile had a positive slope (*p* < 0.05).

### Unknown Initial Angle Trials

Participants’ mean error with the Voice Coils was 0.86 units (*σ* = 0.39) and 0.65 units (*σ* = 0.34) with Vibrotactors. A one-way repeated measure ANOVA showed no difference in the mean of the two feedbacks for these trials, *F*(1,7) = 1.32, *p* = 0.2875. Time to complete trials was 11.7 s (*σ* = 8.06) for Voice Coils and 10.92 s (*σ* = 6.16) for Vibrotactors.

## Discussion

### Angle Targeting Accuracy

The primary result of the study was the comparison of participants’ mean targeting errors for the four feedback types. The results indicated that both Voice Coil and Vibrotactile Feedback resulted in less error than the two no feedback cases. Comparing the mean of both no feedback cases (34.5°) to the mean of the feedback cases (18.2°), the use of feedback reduced targeting errors by 47%. Using feedback, participants working in the discretized workspace were within ±7.5° of the target in 50% of trials and within ±22.5° of the target in 80% of trials. If an increased resolution were desired, there would need to be more variations in tactor frequency, but this could result in more errors due to the difficulty in differentiating between smaller changes in stimulation frequencies. Currently, it is unclear what level of feedback resolution is required to effectively operate a prosthesis and is an area for additional study. The 11 unit workspace of ±90° used in this study was chosen to mimic the full potential range of motion of wrist flexion and extension. Studies need to be conducted to determine if the range (-60° to 60°) and resolution of feedback (±7.5°) are enough to have effective control of the wrist.

### Step Size and Ending Position Effects

The analysis of step size showed that feedback was especially helpful in reaching targets that were further away from the starting point which can be seen by looking at the relative errors for various step sizes. As the step size increased, relative errors decreased showing feedbacks benefits for large step sizes. Looking at errors dependent on target position showed that in general targets ending with stimulations closer to the wrist resulted in less error, especially for Voice Coil Feedback. This agrees with the theory that stimulations are easier to recognize when close to anatomical landmarks [28], in this case the wrist. The stimulations closer to the elbow become harder to distinguish since they were not close to a landmark. Instead of using uniform center-to-center spacing, it could be more useful to space the tactor closest to the elbow further from the middle tactor than the one closest to the wrist, however this comes at the cost of covering more space on the forearm.

### Learning in the Study

In regards to learning, the participants achieved the lowest targeting errors when the feedback was second for that participant and both of the highest targeting errors occurred when the feedback was first. However, the regressions and order of appearance of the feedback did not show significant results. One exception was the participant who achieved the lowest targeting error with Voice Coils as the second feedback, also tied for the lowest errors with Vibrotactors as the first feedback. It may have been that to see more improvement with feedback more trials were needed on the same day or perhaps over multiple days. Since there were 11 different feedback stimulations and 9 targets, participants may have had insufficient trials with feedback to significantly improve. The lower bound for errors shows the potential for feedback where the lowest error in terms of degrees was 4.9° for Voice Coils and 8.9° for Vibrotactors.

### Unknown Angle Trials

The unknown position trials showed that participants were still able to accurately reach the target position without knowing where the virtual wrist started and in about the same amount of time as the known initial position trials. Additionally, targeting errors were lower in this case than the known initial angle trials, which is likely due to target positions being only 0°, ±30°, and ±60° and potentially learning from previous trials. The objective of these set of trials was accomplished which showed that participants could accurately reach target positions without the need for knowing initial positions. This would be useful for an amputee since knowing the initial position would require examination of the prosthesis which is undesired since the objective of the device is to reduce visual attention as much as possible.

### Comparison of Results to the Literature

The results of the study show the advantages of feedback to no feedback; however, they will also be compared here with 3 other similar studies: [6, 12, 16] which were discussed in the introduction section. All numbers from other studies are estimated from figures in the studies since exact data was not available. Compared to [6], this study reports slightly larger mean participant targeting errors compared to skin stretch (12°) and no feedback (16.5°), although the reduction in error from no feedback was 47% in this study whereas the reduction in [6] was closer to 28%. The lower mean targeting errors in the study were likely due to the use of only 3 target positions as compared to 9 positions in this study. The importance of this comparison is that this study has much less errors over no feedback with an increased amount of targets. In comparison to the skin stretch results in [16], which had about a 40% reduction in errors from no feedback, this study also performed better. Mean targeting errors were higher in this study (1.1 units vs. approximately 0.6 units), although fewer targets (5) were used in their study and sEMG was not used as the control.

It is interesting to note how the reductions in no feedback were so different between [6] and [16] in which both used similar skin stretch feedback. It is possible that the use of sEMG control in [6] over using a force sensor controlled by the fingers and thumb in [16] contributed to this. Similarly in [12] they used a mouse to move the virtual hand prosthesis instead of sEMG; however, this study found it necessary to use sEMG to model the control of a prosthetic as much as possible. Comparing mean participant discretized targeting errors to [12] (feedback without touch feedback) showed that feedback in this study and theirs were similar, although the Voice Coils had a 45% success in reaching the target and 80% in one deviation whereas their study reported about 35% and 70% for vibrotactors. The main point here is that results were similar with those in this study being slightly better and also with an increased workspace (11 unit workspace, 9 targets compared to 8 unit workspace, 8 targets) and they used eight tactors with “on/off” feedback compared to the three here with a feedback scheme.

Additionally, the median time for Vibrotactile and Voice Coil Feedback was approximately 10 seconds in this study whereas Electrotactile and Vibrotactile Feedback in their study took 50 or more seconds with feedback. Participants in our study only took about 10 seconds to complete trials while they took about 50 seconds in the study of Witteveen et al. Although Witteveen et al. used a slightly different experimental protocol since the hand was given a spring constant and participantsâ?? needed to hold the hand in place for 2 seconds to end the trial, still it is not likely that this alone would explain the large time discrepancy. It is also interesting that our participants took about the same time to complete trials even when they did not know the initial position as in the main 32 trials. This is important since it shows that participants were not just counting changes in feedback but could instead accurately differentiate between the 11 discrete events.

It is worth noting that for the comparisons made here that our study used strict controls (sEMG control along with no visual or audio feedback) while only the work of Wheeler et al. used sEMG and none had controls on visual or audio feedback. We propose that studies such as these should use noise and visual canceling of feedback which was not employed in the three studies compared here which could increase errors in these studies if included. With visual and audio cues available to the participant, it is not possible to effectively evaluate the performance of haptic feedback.

### Benefits of Normal Stimulation and Future Improvements

It can be seen from this study and others that the main forms of stimulation are through low frequency skin stretch, vibrotactors, and normal stimulation devices. The advantage of vibrotactile and normal stimulation over skin stretch is that when the hand is at rest, the tactors can stop stimulating the skin to relay proprioception and then resume the stimulation when the joint is moved. For a rotational skin stretch device, the device would have to stay stretching the skin or go to a no-stretch position and then re-stretch the skin back to that position when the joint moves which could create delays in the feedback. Additionally, skin stretch devices are currently much larger and heavier than vibrotactors or the prototype presented here. The prototype used in this study has an advantage over vibrotactors in that it operates at a lower frequency range than vibrotactors. Operating at a lower frequency may be more comfortable for the user since at high frequencies the buzzing sensation may not be comfortable. The device also generates less noise than vibrotactors which could be beneficial for daily use.

A note about the device used in this study is that it was a prototype and further improvements in the device and feedback scheme can be made. The feedback device might be further optimized for producible force or modifications to its size for incorporation into a hand prosthesis system. For the feedback scheme, the range of 5 Hz–100 Hz stimulation frequencies was chosen since 5 Hz was deemed the minimum frequency for information transfer and 100 Hz was used to maximize the frequency range. While we deemed 2 Hz to be too slow for information transfer, in [17] good discrimination of a normal stimulation device in the range of 2–8 Hz was found indicating that a lower frequency might increase accuracy in recognizing a feedback pattern. More testing is required to determine if the device could be operated in a narrower frequency band to reduce the highest frequency and thereby improve user comfort. Additionally, if more resolution was desired more discrete changes in stimulation frequency could be used; however, eventually this will approach a continuous pattern which might reduce feedback recognition.

## Conclusion

This study presented the testing of a normal stimulation haptic prototype device combined with a novel haptic feedback scheme in an experiment in which participants controlled a 1DOF virtual wrist prosthesis. The feedback scheme is unique in that it intelligently combines stimulations from three different tactors to relay discrete changes in position information. Normal stimulation had significantly lower errors over no feedback cases and generally had improved performance over shear stimulation. In comparison to other studies normal stimulation had similar results while covering a larger workspace and only using a set of three tactors compared to a similar study that used eight. Additionally, participants were able to achieve target positions quickly and without need for knowing the starting position. The results and analyses presented in this study could be used for the design of improved feedback schemes to improve the accuracies achieved here. The high accuracy achieved in this study warrants the exploration of normal stimulation devices and the feedback scheme presented.

While the experiments in this paper were one-dimensional, the device has the potential to be extended to two-dimensional movements using the cross-pattern of nodes used in the pilot study. A single DOF could be controlled with the tactors going from the wrist to the forearm, while the second could be controlled with those going across the forearm. Extension to 2DOF should not see any suffering in performance based on the high recognition of stimulation sites as shown in our pilot study. If multiple-DOFs were to be controlled, a grid of tactors could be used to control up to 3DOF, although this would need to be tested to see if it is feasible. Further testing could include direct comparison to on/off feedback schemes, including cognitive load, and use with a prosthetic user to examine its true effectiveness. Further testing of the type of feedback presented here will be necessary in bringing a viable sensory feedback device to upper limb amputees. Although this study was targeted for use with a prosthesis, the prototype and feedback scheme need not be limited to relaying information to a prosthetic user and could also be beneficial in other haptic applications.

## Supporting Information

S1 Dataset Experimental results data files. (Tab 1) Results of ANOVA for comparing all feedback types for the stated cases. (Tab 2) Results of repeated measure ANOVAs for comparing all feedback types for stated cases. (Tab 3) Trial completion times. (Tab 4) Unknown starting position trial completion times.
(XLSX)
